# Flexibility Training and Functional Ability in Older Adults: A Systematic Review

**DOI:** 10.1155/2012/306818

**Published:** 2012-11-08

**Authors:** Liza Stathokostas, Robert M. D. Little, A. A. Vandervoort, Donald H. Paterson

**Affiliations:** ^1^Canadian Centre for Activity and Aging, School of Kinesiology, The University of Western Ontario, London, 3M Centre 2225, ON, Canada N6A 3K7; ^2^School of Physical Therapy, Elborn College 1400, The University of Western Ontario, London, ON, Canada N6A 3K7

## Abstract

*Background*. As indicated in a recent systematic review relating to Canada's Physical Activity Guidelines for Older Adults, exercise interventions in older adults can maintain or improve functional abilities. Less is known about the role of flexibility in the maintenance or improvement of functional abilities, and there currently does not exist a synthesis of the literature supporting a consensus on flexibility training prescription. *Purpose*. To systematically review the effects of flexibility-specific training interventions on measures of functional outcomes in healthy older adults over the age of 65 years. *Methods*. Five electronic databases were searched for intervention studies involving concepts related to aging, flexibility, functional outcomes, and training interventions. After evaluating the articles for relevance, 22 studies were considered. * Results*. The results suggested that while flexibility-specific interventions may have effects on range of motion (ROM) outcomes, there is conflicting information regarding both the relationship between flexibility interventions and functional outcomes or daily functioning. *Conclusions*. Due to the wide range of intervention protocols, body parts studied, and functional measurements, conclusive recommendations regarding flexibility training for older adults or the validity of flexibility training interventions as supplements to other forms of exercise, or as significant positive influences on functional ability, require further investigation.

## 1. Introduction

As indicated in a recent systematic review relating to Canada's Physical Activity Guidelines for Older Adults, exercise interventions (comprised of aerobic and strength training) in older adults can maintain or improve functional abilities [1]. Less is known about the role of flexibility in the maintenance or improvement of functional abilities. While joint flexibility may decrease with age, with the potential to affect normal daily function, older adults do maintain the ability to improve flexibility through stretching exercises [1]. The 2009 American College of Sports Medicine (ACSM) position statement “Exercise and Physical Activity for Older Adults” [2] noted there is a lack of studies of the effects of range of motion exercises on flexibility outcomes in older populations and a lack of consensus regarding the prescription of stretching exercises for older adults. Despite the lack of a synthesis of the literature to support the recommendation of the inclusion of a flexibility component to older adult exercise programs, many older adult activity programs place a considerable emphasis on flexibility. Stretching exercises are used extensively in the rehabilitation context wherein injury or disease may have resulted in a restricted range of motion specific to given joints, and the goal is to regain “normal” range of motion [3]. However, the present paper is focused not on stretching exercise for rehabilitation purposes but for the role of flexibility in general exercise prescription for older adults.

In light of the significant benefits of an exercise program for an aging population, it is important to provide evidence-based prescription for older adult exercise programs and highlight areas of research requiring further investigation in order to maximize these benefits. The goal of a flexibility program is to improve range of motion in the major muscle-tendon groups in accordance with individualized goals [4]. For the majority of the aging population, the goals may not be related to athletic performance, but rather performance of functional abilities in activities of daily living. Nevertheless, there is relatively little research on the potential benefits of flexibility-specific training interventions for this population in that context. Despite the lack of research and no “known health benefits” [5], again, there is still a tendency in the literature to mention flexibility training as a presumed “component of fitness” and beneficial adjunct to other forms of exercise. Therefore, the purpose of this systematic review is to investigate the functional outcomes of flexibility specific training in older adults.

## 2. Methods

### 2.1. The Literature Search and Inclusion Criteria

A search strategy was developed, where all reasonable expressions of the concepts of aging, flexibility, functional outcomes, and training interventions were considered (see Appendix for a sample search strategy). A comprehensive electronic literature search was conducted on five online databases: PubMed (NCBI; 1950-), Embase (OVID; 1974-), CINAHL (OVID & EBSCO; 1982-), Scopus (1823-), and SportDiscus (EBSCO; 1800-). The literature was searched up to January 2011. The final inclusion criteria for this paper were (1) an original research article, (2) human subjects, (3) an intervention study, (4) flexibility training was an independent intervention or was used as a control, (5) aged population (mean age ≥ 65 years), and (6) the population was healthy but allowing for arthritis, osteoarthritis, and those residing in assisted living (based on age and risk, not diseases or other medical conditions). For this paper, healthy was operationally defined as community-dwelling and assisted living with the health and function and cognitive ability to participate in light physical activity interventions and complete physical function measures. Interventions targeting specific chronic conditions (aside from arthritis and osteoarthritis) were excluded from review. Despite their use in flexibility training, tai chi- and yoga-based studies were excluded from this paper because by nature they include strength components. The electronic search yielded 4037 citations. The citations and applicable electronic versions of the article (where available) were downloaded to an online research management system (RefWorks, Bethesda, MD, USA).

### 2.2. Screening

Two reviewers independently (RL, LS) evaluated the articles for relevance using standard systematic review methodology leading to further consideration of 22 articles.

Two reviewers independently completed standardized data extraction forms for each level of screening. Three levels of screening were utilized. Level 1 screening was based on article titles, Level 2 was based on the title and abstract, and Level 3 was a full text screening. The articles that progressed through to Level 3 were retrieved electronically or manually via the Canadian interlibrary system and were printed from electronic copy. Any cross-referenced articles from the reference section of Level 3 articles were hand-screened. Disagreements regarding inclusion were resolved through discussion with a third reviewer (DP).

### 2.3. Data Extraction

Data from the included studies were extracted (Table 1) and organized by the target muscle groups of the flexibility interventions. Two reviewers completed standardized data extraction forms. One reviewer performed the data extraction for each paper assigned to them and the extraction was verified by another reviewer. The reviewers were not blinded to the journal or the author names when extracting information from the articles.

### 2.4. Level of Evidence

The approach used to establish the level and grade of evidence was consistent with Lau et al. [6] which provide predefined and objective criteria. Thus, the strength of the evidence was assessed for flexibility interventions and functional outcomes in older adults with respect to general recommendations and appropriate dose.

### 2.5. Quality Assessment

Quality assessment of the included studies was also performed (Table 1). The Downs and Black [7] scale was selected to assess the quality of each study as it is appropriate to evaluate nonrandomized investigations, and it contained the highest number of relevant items for the needs of this paper. However, as not all items were relevant to the various study types included in this paper, a modified version of the checklist was employed for each of RCTs (randomized control trials), and non-RCTs study types. Thus, the quality of each study was also established similar to the method of Gorber et al. [8] to include the most relevant components of the scoring tool. Therefore, a modified version of the Downs and Black checklist was used with the final checklist consisting of 22 items with a maximum score of 24 points for the studies of a RCT design; 22 items for non-RCT designs with a maximum score of 23; experimental single group interventions had a maximum score of 18 from 18 items; experimental single-group and single-session studies were based on 14 items for a maximum score of 14. Higher scores reflected a superior quality of investigation.

### 2.6. Integration of Findings

Due to the heterogeneity across study populations, methods used, and outcomes assessed, we conducted a narrative synthesis of the results.

## 3. Results

### 3.1. Description of Studies

**Figure 1:**
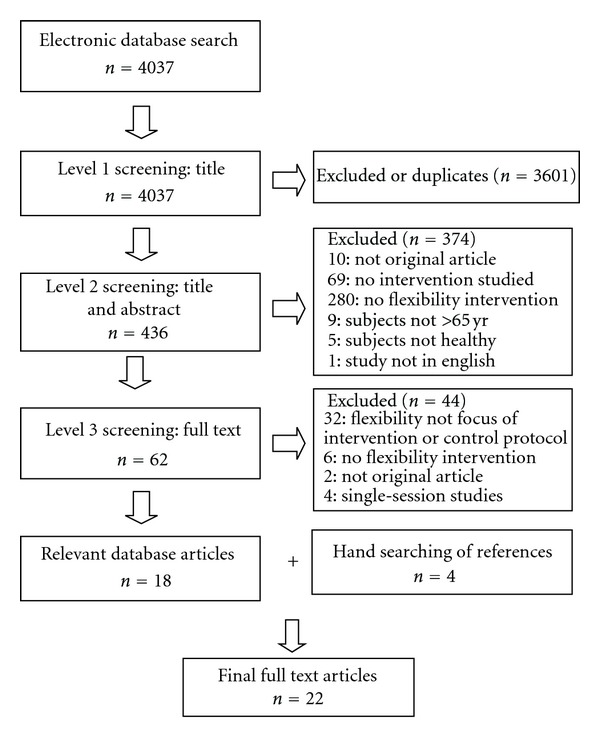
Article Screening Flow Chart.

The initial search yielded 4037 articles. Twenty-two articles were ultimately included after meeting Level 3 inclusion criteria (Figure 1). Of the final 22 articles, 18 were from electronic database searching, and 4 were found by hand searching. Quality assessment indicated that the RCT studies (*n* = 13) were of good quality with an average score of 18 out of 24. The non-RCT studies (*n* = 6) had an average score of 14 out 23. An average score of 12 out of 18 was assessed for the experimental single-group studies (*n* = 3). Fourteen articles were conducted in the United States [10–12, 14, 15, 17, 18, 20, 22, 25–29], while the remainder of the studies were from Japan [19], Brazil [23, 24], Turkey [21], Australia [9, 16], Taiwan [30], and Canada [13].

### 3.2. Population

The mean sample age was 74.1 years, ranging from 64 years [30] to 88.8 years [14]. Seven studies included populations that were ≥80 years of age [10, 11, 13, 14, 18, 25, 27]. The number of participants in the articles of this paper ranged from 7 [27] to 132 [30]. There were a total of 1127 participants, 841 of whom were female (75%), while 286 were male. Six studies were female only [15, 23–26, 29]. Twenty studies were based on community-dwelling populations, and two studies involved individuals residing in assisted-living facilities [10, 13].

### 3.3. Outcome Measures

Outcomes were measured using flexibility measurements, physical ability tests, and questionnaires. One study also used brain imaging for the purposes of identifying changes in hippocampal volume with training [20]. A common outcome measure was simply whether there was a change in range of motion usually assessed by goniometry [15, 17, 20, 24–28, 30]. The inclusion of these studies in the review (although they reported no “functional outcome”) was to provide the data for the purpose of determining whether older adults would, in fact, improve range of motion about different joints with various flexibility exercise programs. Functional outcomes were operationally defined as tests or measures designed to reflect abilities for various levels of daily activities and potentially related to maintenance of independence of older adults. In general, these tests assessed ability in a function that involved more than a single fitness component (e.g., not just a strength measure of a weight that could be lifted, but rather a performance that may involve strength and power as well as balance and agility). The most commonly used tests of functional outcome were gait and various walking speeds [11, 13, 14, 19, 21, 22, 26], the sit-and-reach test [10, 12–14, 19, 29], the sit-to-stand test [9, 12, 14, 16, 18, 19], functional reach test [9, 10, 21, 26], step test [9, 16], timed up-and-go (TUG) [10, 13, 14, 16, 19, 24, 26], and Romberg test [11, 21]. Other functional outcome measures were Berg balance scale [13], questionnaires [9, 12], peg board [11], red-light-green-light [11], Lequesne's index of disability [30], the physical performance test (PPT) [11], and the gallon jug shelf test [14]. Gait and walking speed proved to be more positively affected by flexibility training than other outcome measures [14, 19, 21, 22, 26], although this was not entirely consistent [11, 13]. Several studies showed increases in flexibility-related outcomes, but lacked significant changes in other more applicable and generic measures of functionality. Only one study followed up on outcome maintenance after the postintervention measurements. This study found that knee torque and timed up-and-go showed improvements compared to the control group, which persisted for four weeks after intervention [24].

In the sub-group containing eight studies of the very old (≥80 years), frail, and assisted-living populations [10, 11, 13, 14, 18, 21, 25, 27], there were significant improvements seen in functional reach [21], sit-to-stand [10, 14] and 30 m walk times [21], but no changes in the PPT [11] and mixed results for flexibility, strength, balance, and TUG tests. As compared to studies on less aged independently-living populations, the outcomes results of this sub-group were similar, except that the more aged/dependent group had less consistent flexibility outcomes; there were some neutral outcomes and some negative changes following flexibility training.

Six out of the 22 studies included several functional outcome measures and were considered most relevant to this paper [11–14, 16, 19], and are individually reviewed herein. Four of the six conducted randomized control trials (RCT) [11–14], and only two studies reported sample sizes less than 68 participants. Brown et al. [11] conducted a 12-week RCT (*n* = 87), wherein the flexibility trained group demonstrated only flexibility measure improvements and no change in functional outcomes. The measures included were very practical and included the chair stand, picking up a penny, putting on and taking off a coat, and the Romberg balance test. However, it should be noted that this study also showed minor strength and balance losses. In a one year RCT, King et al. [12], similarly, showed no change in most functional outcomes in the flexibility trained group. Functional measures included lift-and-reach, sit-to-stand, sit-and-reach, self-rated physical performance, self-efficacy for physical performance scale, and perceived functioning and well-being. There were only significant increases for the sit-and-reach test in men only (10.4 to 11.9 inches) and decreases in self-rated daily bodily pain scores (by 7.3% for women and 9.4% for men). Lazowski et al. [13] conducted a 16-week RCT (*n* = 68). Timed up-and-go increased (worsened) from 27 to 33 seconds, and no changes were seen in any other functional measures or in the sit-and-reach test. Additional functional measures included strength tests, Berg balance scale, self-paced and fast-paced walk tests, stair-climbing, and the functional independence measure for functional capacity. Stanziano et al. [14] employed an 8-week RCT (*n* = 17), where the experimental flexibility group significantly improved in every measure: chair stand repetitions (11 to 13), modified ramp power (69 W to 86 W), arm curl repetitions (12.9 to 18.8), gallon jug shelf test (13.4 to 11.5 seconds), 8 foot timed up-and-go (8.7 to 7.6 seconds), and 50 foot gait speed (13.9 to 12.3 seconds). The study by Bird et al. [16] was a 32-week randomized cross-over design (*n* = 32) with 16 weeks spent in the flexibility group. Four functional outcomes improved significantly for the flexibility group: TUG (7.6 to 6.6 seconds), sit-to-stand (22.6 to 18.0 seconds), step test (13.5 to 17.6 steps), and mediolateral sway range (eyes open: 4.16 to 2.97 cm; eyes closed: 6.87 to 5.64 cm). Strength was also tested, with no improvement by the flexibility group. In a 12-week non-RCT (*n* = 117), Takeshima et al. [19] reported no improvements for the flexibility trained group in the 12-minute walk, arm curls, chair stand, TUG, functional reach, back scratch, and sit-and-reach. These six studies exemplify the mixed results of the functional outcome measures in this paper. They serve as a strong representation of the lack of consistency of functional outcomes in the literature and therefore, any specific recommendation regarding type or frequency of stretching exercises is premature.

Overall, seven of 22 studies demonstrated mostly positive functional outcomes [9, 10, 14, 16, 21, 23, 29], while six reported mostly negative functional results, that is, no improvement in variables [11–13, 18, 19, 22]. Ten studies showed no functional outcome measures [15, 17, 20, 24–30] and only reported changes in flexibility and ROM (although they purported to relate flexibility to function, and were included for their results related to the ability of older adults to improve flexibility). There were no obvious differences between the characteristics of the studies with positive, negative, or no functional outcomes.

### 3.4. Intervention Characteristics

Twelve studies used flexibility training as the sole intervention [10, 11, 14, 17, 21–27, 29], four studies used flexibility training as a significant part of an intervention protocol [15, 16, 19, 30], five studies used flexibility as a control group to compare with various other exercise interventions [9, 11–13, 18, 20], and four studies utilized flexibility exercises along with strength or aerobic exercise in the intervention protocol but in the control group, the entire exercise protocol was flexibility exercises [9, 11, 13, 20]. The types of flexibility training methods varied from simple static stretches (19 studies) to different proprioceptive neuromuscular facilitation (PNF) techniques (1 study). Passive static stretching was the most common method used [9, 11–13, 16, 18–29], while passive static stretching with added weights was used once [17], active-assisted (AA) was used twice [14, 30], active and passive were used in conjunction once [15], contract-relax (CR) PNF was used once [30], contract-relax-agonist contract (CRAC) PNF was used once [30], and hold-relax-agonist contract (HRAC) PNF was used once as well [30]. One study compared multiple methods with each other [30]. Active-assisted stretching had positive and sometimes significant improvements in several outcome measures as compared to the inactive control group, but less significant than the improvements seen with the PNF techniques [30]. Weighted flexibility exercises were similar to nonweighted exercises in one study [15], but significantly better than nonweighted exercises in another [17].

Thirteen studies involved whole body flexibility training [9–21], two focused on hips and calves [22, 23], one focused on hamstrings [24], three focused on calves [25–27], one focused on hip flexors [28], one focused on the trunk [29], and one study focused on the quadriceps muscles [30]. Whole body flexibility training showed some minor to significant increases in outcomes; however, most increases were seen in ROM and flexibility measures, per se, and not in other functional outcome measures. These results were consistent with the overall effects of specific isolated stretching interventions on outcome measures for the related specific body parts.

The mean length of the included studies was 14.2 weeks, ranging from 4 weeks to one year [12, 20]. Results did not differ significantly throughout the range of intervention durations. In the three studies of at least 25 weeks in duration [12, 15, 20], no functional outcome measures were improved other than flexibility and ROM. In the four studies with durations of 6 weeks or less [23–25, 27] TUG improved from 8.4 to 7.2 seconds [24], walking velocity increased 1.07 to 1.22 m/s [23], and flexibility and ROM were improved overall.

The mean frequency was 4-exercise sessions per week, ranging from the lowest frequency of twice per week [9, 10, 12, 22, 24] to 14x/week [27, 28]. Two studies did not report exercise frequency [20, 22]. Some results from these studies include a TUG time decrease from 8.4 to 7.2 seconds [24] and a no-change [10], sit-to-stand time decreased from 10.2 to 9.2 seconds [9] and 9.3 to 7.9 seconds [10] and a no-change [12], a step test increased in reps from 16.5 to 20.2 [9], and an increase in freely chosen gait speed from 1.23 to 1.30 m/s [22]. The 5x/week and 14x/week studies did not include functional outcomes similar to the twice weekly studies. The 3x/week studies included results such as an improved TUG time from 7.6 to 6.6 seconds [16] and a worsening time from 26.8 to 33.0 seconds [13], a functional reach improvement of 16.0 to 19.6 cm [21] and two no-changes [19, 26], a sit-to-stand improvement of 22.6 to 18.0 seconds [16], a step test improvement of 13.5 to 17.6 repetitions [16], a 10 m walk time improvement from 6.44 to 5.99 seconds [26], and a 30 m walk time improvement from 28.1 to 20.0 seconds [21]. Although limited in number, these results show that the frequency of the flexibility training interventions had no noticeable differences compare with 2 and 3 times per week.

The mean flexibility exercise session time was 32 minutes, ranging from 30 seconds [29] to 85 minutes [19]. 

## 4. Discussion

There are currently scientific discussions regarding the utility of stretching exercises which are regularly recommended and conducted as a part of preexercise protocols to reduce injury and increase performance. Earlier reviews of stretching and flexibility have questioned their value in terms of injury prevention, delayed onset of muscle soreness, and improvement of performance [31, 32]. In fact, what occurs physiologically with stretching remains unknown [32]. Due to equivocal evidence thus far, the current American College of Sports Medicine's guidelines for exercise testing and prescription [33] recommended the removal of static stretching as part of a warm-up routine for strength and power activities. Additionally, based on available evidence, the 2011 ACSM position statement for guidance on prescribing exercise suggests performing flexibility after cardiorespiratory endurance or resistance exercise for general fitness programs. This position stand highlighted the need for further research to ascertain the effects of various flexibility prescriptions for various activities and performance goals. From the present review, there is not enough consistent evidence to make recommendations for any specific prescriptions of type, frequency, duration, or length of program related to flexibility training; particularly no specific recommendations can be made regarding the program or dose response of flexibility training to the focus of the present study, the transfer of flexibility gains to functions of daily life, or ability to live independently. A recent systematic review of 106 articles relating the effects of pre-exercise acute-passive static stretching on maximal muscle performance provided 74 methodologically sound studies providing 104 findings [34]. This paper showed that 50% of the 104 findings reported significant reductions in task performances, and the authors concluded that static muscle stretches totaling less than 45 s can be used in pre-exercise protocols without significant decrement to strength, power or speed type tasks; thus a conclusion was to recommend stretches held for at maximum 45 s to avoid loss of strength. Shrier [35] had also previously reported the potential negative acute effects of stretching on performance, but additionally reviewed the literature regarding regular stretching on performance which indicates that regular stretching improves force, jump height, and speed performance. Both reviews recommend further synthesis of the literature with respect to the effects of other forms of stretching on various performance measures.

The difficulties of the ability of this paper to provide a consensus on flexibility training prescription for healthy older adults include the lack of well-conducted studies focused on flexibility in older adults and the lack of consistency in the flexibility protocols employed, functional outcomes measured, and functional results observed. As such, according to the criteria used to assess level of evidence, to recommend stretching/flexibility exercises as a routine component of an exercise program for older adults to enhance health or functional abilities is Level 4, Grade C. The more influential studies in this paper (based on focused flexibility protocols with clear functional outcomes and relatively large sample sizes) [11–14, 16, 19] showed very comparable effects to the overall outcomes of the 26 studies, namely, an ambivalence in the value of flexibility training on functional outcomes that may be related to maintenance of independence in daily activities of older adults. Of these six studies, 5 were RCTs with an average quality assessment score of 18 out of 24. Only two of the six showed improvement in flexibility and functional outcomes ([14] 16/24; [16] 19/24).

In the sub-group (≥80 years), frail, and assisted-living populations there were significant improvements seen in functional reach, sit-to-stand, and 30 m walk times, but no changes in the PPT, and mixed results were observed for flexibility, strength, balance, and TUG tests. This sub-group was similarly ambivalent in the role of flexibility training with functional outcomes to the rest of the study populations, although this group had less consistency in the flexibility-related outcome measures. Frequency and duration differences between studies showed no noticeable differences in outcomes. When different muscle groups were targeted, the flexibility outcomes were expectedly fairly body-part specific. Regarding the different flexibility training methods, active-assisted (AA) stretching had positive and sometimes significant improvements in several outcome measures as compared to the inactive control group, but less significant than the improvements seen with the PNF techniques. Weighted flexibility exercises were similar to nonweighted exercises in one study, but significantly better than nonweighted exercises in another. One study showed ACR-PNF to be much more effective than CR-PNF and static stretching for both ROM and EMG activity. The overall results point to PNF stretching being more effective than non-PNF techniques for improving flexibility outcomes, but not necessarily functional outcome measures.

While flexibility training interventions synthesized in the present paper have been shown to increase flexibility and joint ROM, no consistent increases in functional outcomes have been observed. Therefore, future studies should consider the relationship that increased flexibility and joint ROM have with functional outcomes to determine if the increased flexibility is beneficial and worthwhile in terms of maintaining or increasing functional capacity for healthy older adults. More research is also needed regarding the relationships between outcome variables (i.e., how one variable such as functional reach would relate to another variable such as the timed up-and-go) and on the relationships between outcome measures and quality of life through self-reported functioning/quality of life questionnaires to best determine the applicability of the outcome measures.

Older adults are less concerned with high performance benefits from increased flexibility and more focused on being safely active and safely performing activities of daily living [36]. Injury and fall prevention are also common motives for recommending flexibility programs to older adults. The 2011 ACSM position statement notes that flexibility training may enhance postural stability and balance when combined with resistance training; however, no consistent link has been shown between regular flexibility exercise and a reduction of musculoskeletal injuries or delayed onset of muscle soreness [4]. However, despite the growing literature describing the relationship of flexibility to injury risk in younger populations [31], there is little research regarding older adults.

## 5. Conclusions

This paper found that flexibility training interventions in older adults are often effective at increasing joint range of motion in various joints, and various functional outcomes can be improved. However, due to the wide range of intervention protocols, body parts studied, and functional measurements, conclusive recommendations regarding flexibility training and functional outcomes for older adults remain ungrounded. As such, a specific prescription of how long to hold a stretch, how many repetitions of each stretch to conduct, and the type of stretches to do, is not determinable at this point. Because there is conflicting information regarding both the relationship between flexibility training interventions and functional outcomes, and the relationship between improved flexibility and daily functioning and health benefits have not been established, future research studies should attempt to address these issues.

While there is a lack of evidence to recommend stretching routines outside of a rehabilitative context, there is no additional health or functional risk of including flexibility exercises. As such, in light of increases in functional outcomes achieved by other exercise modes (balance, aerobic exercise, and strengthening exercises), stretching exercises can be included as an adjunct to the above, but the current literature would indicate it would add little to the functional benefits of the other exercise modes. Of note, the evidence-based and expert consensus statements of “Physical Activity Guidelines for Older Adults” of the US, the UK, Canada, and the World Health Organization (Global recommendations) have not included flexibility as a component in the recommendations.

## Figures and Tables

**Table 1:** Table-Flexibility training studies examining the relationship between flexibility and functional abilities in older adults.

Publication country/study type	Objective	Population	Methods	Outcomes	Comments and conclusions	Quality
Barrett and Smerdely, 2002 [9]. Australia. RCT (single-blinded, assessor). **Focus** Major muscle groups.	To determine whether a resistance training program could improve strength, mobility, and quality of life of community-dwelling elderly people versus flexibility control group.	*n* = 40. Age: 67 yr. 10 males, 30 females. Experimental *n* = 20. Age: 64 ± 3 yr. 5 male, 15 female. Control *n* = 20 Age: 70 ± 3 yr. 5 male, 15 female. **Inclusion criteria** >60 yr, healthy.	Prepost 10 wks. Both groups attended class 1 hr, twice wk^−1^, 10 wks. **Intervention** 5 min warm-up including stretching, 8–10 resistance exercises (~45 min) with free weights for both upper and lower limbs, followed by 5 min of stretching. **Control** Stretching of major muscle groups (25 min) light cardiovascular (20 min) and low intensity strengthening (15 min). **Assessment** Isometric strength bilaterally with hand dynamometer: biceps, quads; time to stand and sit 5 times for leg strength. Balance: Functional reach test and step test. Quality of Life: SF36 Health. Survey self-reported. **Analysis** Independent sample *t*-tests. Gender: Chi-square tests. Bonferroni adjustment.	Progressive resistance training group improved significantly in all physical measurements. Flexibility group improved significantly in sit-to-stand (9.6% from 10.2 to 9.2 s) and step test (23.5% from 17 to 20 steps) only. Progressive resistance training had a greater effect than flexibility training on quadriceps strength, left biceps strength, functional reach test and step test. Neither group improved significantly in any subscale of quality of life.	Progressive high intensity resistance training produces greater strength, balance, and gait improvements than a nonspecific flexibility group.	13/18

Klein et al. 2002 [10]. USA. Prospective two-stage intervention. **Focus** Major muscle groups.	To examine the impact of PNF on physical function in an assisted- living population by assessing ROM and isometric strength.	*n* = 14. Age: 87 ± 6.5 yr. Male = 2. Female = 12. **Inclusion criteria** ≥65 yr, no neurological or cognitive impairments, resting BP < 160/100 mmHg, no limiting cardiorespiratory condition or recent surgery Living in assisted-living facility, frail.	Baseline (T1), pretraining (T2, 5 wks), posttraining (T3, 10 wks). **Pre-training** 1 wk^−1^ visit with trainer to increase rapport and interest in participation. **Training program** 40–60 min, 2 wk^−1^Warm-up, cool down, and flexibility (single set 15–20 min, later 2-3 sets) Flexibility: 8 exercises using passive contract-relax PNF technique (6 s isometric contraction then passive stretch held for 20 s then 20 s rest). Hamstrings, gluteals, shins, calves and back). **Assessments** Isometric strength (dynamometer), flexibility (bubble inclinometer for shoulders, hips, and ankles, sit-and-reach for spine, functional-reach for shoulder). Mobility: get-up-and-go test, 5-sit-to-stand. **Analysis** Listwise repeated measures, univariate ANOVA, paired *t*-tests, and Bonferroni adjustment	*n* = 11 Statistically significant differences in 6 of 18 variables: sit-to-stand decreased significantly from 9.33 to 7.91 s (*P* = 0.42). No change in balance, get up and go, single leg stand. Ankle-flexion ROM decreased (improved) from 26.25 to 20.27° (*P* = 0.009). Shoulder-flexion ROM increased from 163.8 to 177.6° (*P* = 0.016) No change in hip flexion, hip extension, ankle extension, functional reach, and sit-and-reach. Significant increases in strength for hip extension and ankle flexion/extension. No change in hip flexion, shoulder extension, and shoulder flexion strength	PNF flexibility training can improve ROM, isometric strength, and selected physical-function tasks in assisted-living older adults. Because the training period was short, 10 weeks, the results suggest that continued training might have a greater impact on physical function and the ability to perform routine daily activities.	13/18

Brown et al. 2000 [11]. USA. RCT. **Focus** Major muscle groups.	To examine effects of low-intensity exercise on factors associated with frailty (gait, flexibility, strength, balance, sensation, response time, and coordination) versus flexibility control group.	*n* = 87. Age: 83 ± 4 yr. **Experimental group (exer)** *n* = 48. Male = 20, Female = 28. Age: 83 ± 4 yr. **Control group (home)** *n* = 39. Male = 17, female = 22. Age: 83 ± 4 yr. **Characteristics** Sedentary, over 78 yrs, living independently but with difficulty. **Inclusion criteria** Medical screening, physical performance test (PPT) for frailty: 17 < score < 32.	Pre-post 3 mo **Intervention** **exer** 22 low intensity strength and flexibility exercise for upper and lower body 3x/week for total of 36 sessions (~3 mo) **HOME** 9 upper and lower body flexibility exercises. Conducted at home (self-report), option to participate on site 1 wk^−1^. **Assessments** *Strength:* physical performance test, isokinetic dynamometer (knee flexors/extensors, ankle flexors/extensors), and hand-held dynamometer (upper extremities) *ROM*: goniometry (shoulders, hips, knees, ankles, and trunk). *Balance:* static (Romberg test), dynamic (balance bean, obstacle course, and gait speed), and weight-shift (Berg balance test). *Gait:* pressure-sensitive foot switches. *Coordination:* Purdue peg board. *Speed of response:* red light to green light, stepping on brake and gas pedals. *Sensation:* Semmes-Weinstein monofilaments. **Analysis** 2 × 2 ANOVA; paired *t-*tests for EXER group only	**Physical performance test** Significant improvements in PPT scores from 29 ± 4 to 31 ± 4; unchanged in control group); improvements were in chair rise, putting on/taking off coat, picking up penny, and Romberg test. **Strength** Significant increases in knee flexor and extensor strength (9% change versus−1% in control) and shoulder abductors. **Range of motion** Flexibility increased in all measurements and in both groups. **Balance** Significant improvements in EXER group for obstacle course, full-tandem of Romberg, Berg balance test, and one-limb standing time. No significant changes in control group **Gait** Significant change in preferred walking cadence in EXER group. **Coordination** Difference between groups was “*almost* significant.” **Response time** Unchanged in both groups. **Sensation** No apparent differences.	The control group lost a small amount of strength and balance in just 3 months, even though flexibility improved. These results suggest that the more comprehensive the exercise intervention, the greater the likely scope of improvement in frailty.	14/24

King et al. 2000 [12]. USA. RCT. **Focus** Major muscle groups.	To evaluate the effects of two different community- based physical activity regimens—on one year physical performance outcomes, perceived functioning and well-being in a sample of community-dwelling, sedentary women and men.	*n* = 103 Age: 70 ± 4 yr Males = 36, females = 67 **Inclusion criteria** >65 yrs, absence of cardiovascular disease or stroke, regularly active no more than 2x/week during the preceding 6 mo, free of musculoskeletal problems that would prevent participation in moderate levels of physical activity.	Pre-post 12 month, 6 month interim assessment 2 exercise classes/week and home exercise at least 2 wk^−1^. Classes one hour, home exercise built up to 40 min sessions. **Experimental group** (fit and firm) Progressive moderate-intensity endurance and strengthening exercises. 5–10 min warm-up, 40–45 min aerobic and strength training circuit, 5–10 min cool-down; target heart rate 60–75% HRR. **Control group** (stretch and flex) Stretching and flexibility exercises. 5–10 min warm-up, 40 min stretching section, 5–10 min relaxation exercises. Stretching for neck, shoulders, back, chest, waist, hamstrings, calves, and hands. **Assessments** *Functional capacity/endurance*: Graded treadmill exercise test (GXT). *Strength and flexibility:* upper body strength (lift and reach task), lower body strength (sit to stand), and flexibility (sit and reach w/Accuflex 1 sit and reach box. *Self-rated physical performance: *a self-efficacy questionnaire. *Perceived functioning and well-being: *scales from the Medical Outcomes Study (MOS) incl. physical functioning, bodily pain, emotional well-being,	(values reported separately for men and women for each group). **Functional capacity/endurance **Submax HR: Fit & Firm had significantly greater improvement versus Stretch & Flex **Strength and flexibility** Lift and reach task: Fit & Firm had significantly greater upper body strength than Stretch & Flex. Sit-to-stand: No significant results. Sit-and-reach: Men assigned to Stretch & Flex had significantly greater increases than men in Fit & Firm. No statistical difference in women, but trend for greater improvement for women in Fit & Firm versus. Stretch & Flex. Women in Fit & Firm had significantly greater increases in flexibility at 12 mo than men. **Self-rated physical performance** Significantly greater increases in walking distance and self-efficacy for heavy lifting in Fit & Firm than in Stretch & Flex. **Perceived functioning and well-being** Only pain scale had significantly greater effects for Stretch & Flex (also statistically significant within group) than Fit & Firm.	Community-based physical activity regimens focusing on moderate-intensity endurance and strengthening exercises or flexibility exercises can be delivered through a combination of formats that result in improvements in important functional and quality of life outcomes.	21/24
			energy/fatigue, sleep problems, sense of mastery, and self-esteem. **Analysis** ANOVA, ANCOVA, MANCOVA, Tukey's studentized range test, and least-squares means procedure.			

Lazowski et al. 1999 [13]. Canada. RCT. **Focus** Major muscle groups.	To evaluate group exercise programs in long-term care versus. flexibility control group.	*n* = 68. Age: 80 ± 0.9 yr. 11 male, 57 female. Functional Fitness for long-term care program (FFLTC) group *n* = 36. 7 male, 29 female. Range of motion(ROM) group *n* = 32. 2 male, 30 female. Residents of five long-term care institutions (>3 month) **Inclusion criteria** No recent cardiovascular events, uncontrolled high BP, recent fracture, total blindness, or deafness. Ability to stand with minimal assistance. Walking devices and wheelchairs allowed.	Pre-post 4 mo **Intervention ** **FFLTC group** 45 min, 3 wk^−1^ warm up/stretching (5 min), walking (15 min), progressive upper and lower body strengthening (10 min), balance, and cool down stretching (5 min). **Control group ** Seated range of motion group vocal exercises, word/memory games, range of motion exercises (fingers, hands, arms, knees, ankles), and relaxation exercises. **Assessments** Mobility-timed up-and-go (TUG). Functional balance-Berg Scale. Stair climbing power. Functional ability-functional independence measure (FIM). Flexibility-Modified sit and reach test. shoulder flexion. Isometric strength: elbow flexion, shoulder abduction, knee extension, hip abduction/adduction. grip strength. Isotonic Strength: knee extensors.	86% and 79% compliance ROM scored lower on several measures at baseline. Considerable variability within all conditions on all measures. FFLTC led to significant improvements in mobility, balance, flexibility, and various measures of strength. Functional capacity was unchanged in the FFLTC group and decreased in the ROM group. No change in grip strength, gait speed and stair-climbing power for either, group. ROM (control). Increased TUG time. No change in balance or lower body flexibility. Non-significant 3.5% change in shoulder flexion ROM. 21% increase in shoulder abduction strength. Decline in isotonic leg and hip strength.	The FFLTC is suitable for long-term care residents, feasible for staff to deliver, and low-cost. Most importantly, functional outcomes clearly superior to seated ROM program. ROM may improve shoulder-abduction strength, but will not prevent declines in lower body strength, mobility, and balance.	21/24

Stanziano et al. 2009 [14]. USA. RCT. **Focus** Major muscle groups.	To examine impact of an active- assisted (AA) flexibility program on ROM and functional performance variables in older persons living in a residential retirement community (RRC).	*n* = 17. Age: 88 ± 5.4 yr. Experimental group *n* = 8. 90 ± 4.5 yr. 1 male, 7 female. Control group *n* = 9. 88 ± 6.2 yr. 3 male, 6 female. **Inclusion criteria**Living in an RRC, ability to sit upright in a chair for 30 min (no akathisia, neurological, or osteoporotic limitations).	Pre-post 8 wks 2 wk^−1^ **Experimental group** 10 stretches: back scratch (shoulder flexion/abduction), standing thigh (hip hyperextension), side lunge (hip abduction), overhead back (shoulder hyperflexion), overhead side (lateral trunk flexion), cross chest (horizontal shoulder adduction), seated trunk twist (trunk rotation), seated hamstring (trunk/hip flexion), and seated calf (dorsiflexion). 10 repetitions, 4-5 s each. **Control group** Arts and crafts class with limited physical exertion. **Assessment** Conducted 1 wk before and after training period. Flexibility: back scratch test (BS). Modified chair sit and reach test (SR). Supine knee extension test (KE). Modified total body rotation test (BR). Strength/power: 30-sec chair stand (CS). Modified ramp power test (MRPT). 30-sec arm curl (AC). Gallon jug shelf test (GJST). Mobility: 50-foot gait speed test (GS). 8-foot timed up and go (UG). **Analysis** ANCOVA	*n* = 13 **Flexibility** Significant increases in ROM made by experimental group for all measures but left side BS and right side SR. Control group showed no change in any flexibility measure but a significant loss in ROM for right-side knee extension. **Functionality** Experimental group significantly improved CS and MRPT, while control had significant declines. Experimental group significantly improved in AC and the GJST, while control had no change. Experimental group reduced time taken to complete the UG and GS.	Eight weeks of AA stretching may be an effective intervention for improving ROM, mobility, and functional power for older persons living in a RRC. Data provide clear link between flexibility and functional performance in older persons and support the inclusion of flexibility training in interventions designed to increase independence in older persons.	16/24

Raab et al. 1988 [15]. USA. Non-RCT. **Focus** Major muscle groups.	Examine the ability of weighted and nonweighted exercises to increase flexibility in older adults in the hip, shoulder, wrist, ankle, and neck.	*n* = 46. Female. Experimental groups Exercise (no weights, EN). *n* = 16. 70 ± 3.9 yr. Exercise (with weights, EW). *n* = 17. 70 ± 3.2 yr. Control group No exercise. *n* = 1371 ± 8.1 yr. Healthy, active older adults.	Pre-post 25 wks. **Exercise program** 60 min, 3 days/week; 5–10 min treadmill warm-up; 10 min aerobics at 65% HRmax; 25–30 min whole body strength and flexibility exercises; 10–15 mon cool-down. Exercises involved active and passive stretching held for 20 s, slow circling motions for ROM, and repetitive movements for example, leg curls. EW had gradual introduction of wrist and ankle weights. **Assessments** Shoulder flexion and abduction. Neck rotation. Wrist flexion/extension. Ankle flexion/extension. Hip flexion. **Analysis** Two-way ANOVA, Dunn-planned comparisons with two contrasts, one-and two-tailed *t-*tests.	Flexibility improved significantly for exercise groups in ankle plantar flexion, shoulder flexion abduction, and cervical rotation to the left. Hip flexion (reflecting hamstring flexibility) increased for all groups, with no between-groups differences. The exercise with no weights groups had nearly 2.5x greater increase in ROM than exercise with weights for shoulder abduction. No other flexibility comparisons in the exercise groups were significant. No functional outcomes.	Exercise can increase flexibility in healthy, older women by improving shoulder flexion and abduction, ankle plantar flexion, and cervical rotation. For shoulder abduction, a nonweighted exercise program can produce greater flexibility gains in older adults than a weighted exercise program, and should be considered if flexibility is the primary goal.	13/23

Bird et al. 2009 [16]. Australia. Randomized Crossover. Tria.l. **Focus** Major muscle groups.	To determine the effect of community-based resistance- versus flexibility-training programs on balance and related measures.	*n* = 32. Age: mean 67 yr. Males = 18, Females = 14. Sedentary. **Inclusion criteria** No history of stroke or other neurological disease or current diabetes, cardiovascular disease, or uncontrolled hypertension. No use of walking aids.	Pre-post 16 wks, 4 wk washout, 16 wks (crossover). **Intervention** Both groups had 3 sessions^.^wk^−1^ for 16 wks, then 4 wk washout, then switched to other group for 16 wks. **Resistance training (RT)** 2-3 sets of 10–12 reps. **Flexibility training (FT)** 40–45 min with 16–20 stretches; two stretches for each of: hamstrings, quadriceps, back, and chest. **Assessments** Balance, force plate. Timed up-and-go. 10 times sit-to-stand. Step test. Lower limb strength (right and left knee-flexion and extension) with an isokinetic dynamometer. **Analysis** Repeated measures ANOVA	Lower limb strength increased significantly in the RT group, but not in the flexibility group and there was a significant difference between the two groups. Significant improvements were seen in both groups for timed up-and-go, 10 times sit-to-stand, and step test. Significant improvements in medial-lateral sway *range* were seen in the flexibility group only. Significant decreases in sway *velocity* were seen in both conditions.	Significant improvements in balance performance were achieved with both resistance-training and standing flexibility-training programs in healthy untrained older adults. Flexibility program did incorporate some degree of balance training in the nature of the flexibility tasks.	19/24

Swank et al. 2003 [17]. USA. Non-RCT. **Focus** Major muscle groups.	To determine the effects of adding modest hand and ankle weights to whole-body stretching exercise on ROM.	*n* = 43. Age: 55–83 yr. Body Recall (BR). *n* = 18. 68 ± 5.6 yr; 8 male, 10 female. BR + Weights. *n* = 14. 68 ± 3.1 yr. 4 male, 10 female. Control. *n* = 11. 69 ± 6.5 yr. 1 male, 10 female. Participants of body recall older adult low intensity flexibility program. **Inclusion criteria**No overt disease or any severely limiting orthopaedic problems	Pre-post 10 wks. **Intervention** Supervised. BR = pain-free, smooth, rhythmic whole-body movements. **Training group 1 (BR)** 60 minutes, 3 wk^−1^. **Training group 2 (BR** + **W)** 60 minutes, 3 wk^−1^with gradual progression of ankle weights and band exercises. **Control group ** *No description. * **Assessment** ROM (goniometer) for neck (left and right rotation), hip (flexion and extension), shoulder (flexion and abduction), knee (extension and flexion), and ankle (plantar and dorsiflexion). **Analysis** ANCOVA, Levene's test of equality. Tukey Honestly Significant Difference test, *P* = ≤ 0.01.	(pre values not given). Significant differences found for 6 of 10 ROM measures: cervical rotation (left and right), hip extension, ankle flexion/extension and shoulder flexion, for both BR and BR + W in comparison to control BR + W showed significantly greater increases in 4 of 6 measures that showed significant change: cervical rotation (left and right), hip extension, and ankle dorsiflexion versus BR.	Found that the addition of weights enhanced effectiveness of stretching exercise. It is likely that a positive effect was noted for 2 reasons: increased resistance during exercise movement and exercises were performed through full ROM. It seems plausible to hypothesize that greater effects would be shown by addition of weights to stretching routines for nursing home clients or free-living, otherwise sedentary elderly.	14/23

Alexander et al. 2001 [18]. USA. Non-RCT. **Focus** Major muscle groups.	(1) Analyze the biomechanics of rise performance during chair-rise tasks with varying task demand in more disabled older adults. (2) To determine whether a strength-training program might improve chair-rise success and alter chair-rise strategy versus. flexibility control.	*n* = 30 (final). Training Group. *n* = 16. Age: 82 ± 6.0 yr. 4 male, 12 female. Control group. *n* = 14. Age: 84 ± 7.4 yr. 2 male, 12 female. Residents of local housing facility. **Inclusion criteria** >65 yr. No lower extremity hemiplegia or amputations, blindness, acute inflammatory or infectious illness, and no dementia. Must complete the easiest chair-rise task. Cannot be currently involved in formal exercise.	Pre-post 12 wks. **Intervention** **Resistance training** 1 hour/day, 3 days^.^wk^−1^ using HydraFitness hydraulic equipment for lower body exercise. Also weighted chair rise and weighted ankle flexion/extension. **Control group** Participated in series of seated neck, trunk, arm, leg, and foot flexibility exercises. **Assessment** Seven chair rise tasks, biomechanics of tasks H = using hands NH = without using hands 60/100/140 are seat heights as % of floor to knee heights). **Analysis** Two-way ANOVA, repeated measures ANOVA with pairwise post hoc comparisons (Fisher's PLSD).	Only training group improved ability to complete the most difficult tasks. Controls maintained performances in general, one or two declined. Only significant decrease was in total rise time for both groups at H-100. Centre of Pressure (COP) increased significantly in both groups in all tasks but H-140 (highest seat height). Knee torques increased for both groups significantly for H-100, H-60, NH-100, and NH-100-F. Mean hip torques increased significantly in controls and decreased significantly in training group in H-60.	Subtle, yet significant changes can be demonstrated in chair-rise performance as a result of controlled, short-term resistance training program.	15/23

Takeshima et al. 2007 [19]. Japan. Non-RCT. **Focus** Major muscle groups.	To compare the effects of a walking- based aerobic program, a band-based resistance program, a stretching- flexibility program, a customized balance program, and a Tai Chi program on functional fitness in a group of community older adults.	*n* = 117. 73 ± 6 yr. 64 male, 49 female. Aerobic (AER). *n* = 13. Resistance (RES). *n* = 17. Balance (BAL). *n* = 15.Flexibility (FLEX). *n* = 16. Tai Chi (T-CHI, Yang Style). *n* = 31. Control (CON). *n* = 25. Healthy sedentary. **Inclusion criteria** No meds for hypertension, HRT. No CHD, no regular physical activity.	Pre-post 12 wks. **Intervention** Supervised** **2 days wk^−1^ (RES, BAL, FLEX, T-CHI) 3 days per wk (AER). All had 10–15 min warm-up 60–70 min of specific exercise: AER-Outdoor walking RES-Progressive elastic band exercises for all major muscle groups BAL-Eyes open/closed, exercise on floor, on foam mats FLEX-15 static stretches for upper and lower body (15–20 s each). T-CHI- standardized 24 forms. **Assessments** Functional Fitness. 30 s arm curl test. 30 s chair stand time. 8 Ft timed up-and-go. back scratch test. chair sit and reach test. 12 min walk test. **Analysis** ANOVA. Wilk's criterion. Kolomogorov-Smirnov test.	Improvement in cardiorespiratory fitness (12 min walk) was limited to AER (16%) RES, BAL, and T-CHI, resulted in improvements in upper and lower body strength and balance/agility. RES showed greatest upper body strength improvement (31%). BAL showed greatest lower body strength improvement (40%). Balance/agility was similar across RES, BAL, and T-CHI (10%). Functional reach, similar improvements for AER (13%), BAL (16%), RES (15%). No significant changes in either FLEX or CON on any measure.	It is recommended that older adults participate in a well-rounded exercise program vs. single mode. RES, BAL, and TAI CHI cross domains not specifically targeted in their design. AER necessitates aerobic-specific activity to improve cardiorespiratory fitness. With FLEX, lack of improvement suggests that further study is needed to explore the effect of flexibility exercise training in older adults.	18/23

Erickson et al. 2011 [20]. USA. RCT. **Focus** Major muscle groups.	To evaluate whether 1 year of exercise training increases the size of the hippocampus and improves spatial memory using moderate-intensity aerobic exercise versus a stretching and toning exercise program.	*n* = 120. Experimental (exp). *n* = 60. Age: 68 yr. 16 male, 44 female. Control (con). *n* = 60. Age: 66 yr. 24 male, 46 female. Community-dwelling, sedentary. **Inclusion criteria** Aged 55–80 yr, no dementia, healthy less than 30 min. of PA in last 6 mo.	Both EXP and CON groups included same 5 min of stretching, both before and after exercise. Program lasted 1 year. **Aerobic exercise condition (EXP)** Progressed from walking 10 min at 50–60% HRR_max_ to 40 min at 60–75% HRR_max_ by week 7, then maintained until program finished. **Stretching and toning control condition (CON) **4 muscle toning exercises, 2 balance exercises, one yoga sequence, and one exercise of choice. Told to exercise at RPE of 13–15 on 20 pt Borg scale. **Assessments** VO_2_ max MRI for hippocampal volume. Computer-based spatial memory task. **Analysis** Repeated measures ANOVA. *t-*tests.	EXP group VO_2_ max increased 7.78% while CON increased 1.11%. EXP group had significant group × time interaction for increased hippocampus size (left + 2.12%, right + 1.97%), while CON group declined (left −1.4%, right −1.43%).	Greater increases in aerobic fitness were associated with greater increased in hippocampal volume, suggesting that larger changes in fitness translate to larger changes in volume. Higher aerobic fitness levels at baseline were associated with better spatial memory. Aerobic exercise-induced increases in BDNF are selectively related to the changes in anterior hippocampal volume. Loss of hippocampal volume in late adulthood is not inevitable and can be reversed with moderate-intensity exercise.	19/24

Ceceli et al. 2009 [21]. Turkey. Non-RCT. **Focus** Major muscle groups.	To determine if performing regular ROM exercises had a beneficial effect on the balance, functional activity, and flexibility of elderly subjects.	*n* = 46. Age: 73 yr. 3 males, 43 females. Group 1. *n* = 25. Age: 74 ± 5.15 yr. 21 female; 3 males. Rest home residents. Group 2 (control). *n* = 21. Age: 72 ± 4.13 yr. Female. Inpatient clinic patients, and sedentary housewives. **Inclusion criteria**>65 yr. Able to ambulate without assistive device. Independent in activities of daily living.	Pre-post; 4 month. **Intervention** Supervised ROM exercises. Supine position with 10 repetitions of each upper and lower extremity joints. 3 wk^−1^, 20 min. **Flexibility** Lateral trunk flexion (right and left) distance between middle finger at rest and in max lateral flexion. Anterior trunk flexion: distance from middle finger tip to the ground. **Balance** Sharpened Romberg (SR) test. One-legged stance test (OLST). Both tests performed with eyes open then closed. **Functional activity.** 30 m Walking Time. Functional reach test. **Analysis**Mann-Whitney *U* test.	Significant increase in Group 1 versus 2 in left (10.76 to 13.32 cm, *P* = 0.035) and right (10.47 to 12.88 cm, *P* = 0.44) lateral flexion. No change in anterior flexion (8.66 to 8.24 cm) 30 m walk time decreased significantly from 28.14 to 20 s (*P* = 0.001) in Group 1. Functional reach increased significantly from 15.95 to 19.6 cm (*P* = .014). No significant difference in balance tests.	Authors stated that participating in daily flexibility group exercise increases ROM and causes some improvement in balance. When compared to a randomly selected hospital applied group, the rest home group has better balance, trunk flexibility and functional ability.	11.5/23

Christiansen 2008 [22]. USA. RCT. **Focus** Hips and ankles.	To examine the effects of a hip and ankle static stretching program on freely chosen gait speed of healthy, community-dwelling older people not active in exercise.	*n* = 37. Age: 72 ± 4.7 yr. **Intervention Group**. *n* = 18. Age: 72 ± 4.7 yr. 3 male, 15 female. Control group. *n* = 19. Age: 72 ± 5.0 yr. 5 male, 14 female. Independently living. **Inclusion criteria** In desired age range, healthy, no joint or musculoskeletal pain that limited movement in past month, no diagnosed gait or balance disorder, no falls history, has not participated in formal exercise during the previous 6 month, and has not used an assistive device for walking.	Pre-post 8 weeks. **Intervention group** Hip and ankle stretching. 2 static stretches held for 45 seconds and repeated 3 times alternating sides; total 9 minutes (540 s)/session. Stretches are standing calf stretch and standing hip flexor stretch. **Control group** Ensured no changes in current physical activity. **Assessments** *Passive ROM:* goniometric measurements of hip (hip extension based on Thomas test position) and ankle. *Gait: *shoes on; two walking speeds. **Analysis** Independent *t *tests or chi-square tests, ICC model 2 and form 1, 2-factor repeated measures ANOVA, separate repeated-measures ANOVA, paired *t *tests, and Bonferroni adjustment.	85% compliance *Gait*: significant increase in freely-chosen gait speed for intervention group (1.23 to 1.30 m s^−1^, +0.7m s^−1^, and *P* = 0.016) versus. control (no change). *joint motion:* peak hip extension and knee flexion increased significantly (59.7 to 66.5°) in intervention group, with no change in control (56.2 to 56.1°). Significant increase in intervention group (7.8 to 11.3°) for passive dorsiflexion, with no change in control. *Other: *No significant changes in stride length or joint angular displacement.	Evidence is provided from the results that joint motion is a modifiable impairment that can be effectively targeted for older people with simple, static stretching home-based intervention.	19/24

Cristopoliski et al. 2009 [23]. Brazil. RCT. **Focus** Hips and ankles.	To determine whether a 4 wk supervised stretching program for lower limbs alters gait kinematics in aged population.	*n* = 28. Female. Experimental Group. *n* = 12. Age: 66 ± 4.2 y.r Control Group. *n* = 8. Age: 65 ± 2.9 yr. **Characteristics** Community dwelling. **Inclusion criteria** Healthy, no gait performance limitations.	Pre-post 4 wks. **Intervention** Experimental group.** **3 sessions^.^wk^−1^. Supervised. 5 min walking warm-up. Hip extensor/flexor muscles, ankle plantar flexor muscles. Static stretches, 60 s each, 4 times. **Control** no activity. **Assessment** Static ROM of hip extension and flexion and ankle dorsiflexion by photography. Gait performance **Analysis** Repeated measures ANOVA, univariate analysis, post hoc Scheffe.	Static range of motion changed significantly (*P* < 0.05) in both hip and ankle joints for the experimental group, no change in the control group. Hip extension (73 to 91°). Hip uniarticular flexors (6.3 to 2.0°). Hip biarticular flexors (7.0 to 2.7°). Plantar flexor amplitude (39 to 48°). Experimental group showed increased step length, higher velocity, and reduced double support time after-training.	Supervised stretching program is effective to alter a number of gait variables. Aged participants displayed gait parameters which were similar to those of young adults.	17/24

Batista et al. 2009 [24]. Brazil. Experimental (Pre-post within subjects). **Focus** Hamstrings.	To determine if an active stretching program increases knee-flexor torque and flexibility, antagonistic torque, and functional mobility in older adults, and whether the possible adaptations remain after intervention.	*n* = 12. Sex: female. Age: 68 ± 6.4 yr. Participants in geriatric revitalization program for at least 12 month. **Inclusion criteria** >60–80 yrs, no vascular, inflammatory or lower-limb musculoskeletal disorders. No uncontrolled hypertension Flexibility deficit ≥20°.	4 wks without stretching (A1) followed by 4 wks of stretching intervention (B), followed by 4 weeks without stretching (A2). Participants were own controls (A1). **Flexibility intervention (B)** 2 wk^−1^; 4 wks. Standing in front of table, therapist aligned spine with bar, participant flexed knees and trunk until hands reached table. Then extend knee and tilt pelvis anteriorly to max painless tension Held for 60 s, returned to standing for 30 s. Repeated 7 times (7 × 60 = 420 s). **Assessments** Knee extension ROM deficit (goniometer). Isokinetic torque of knee flexors and extensors. Timed Up-and-Go (TUG), functional capacity. **Analysis** Repeated one-way analysis of variance. Student's Newman-Keuls test.	Significant decrease in knee extension deficit after intervention (24.1° to 14.1°, *P* = 0.0001), but not completely maintained after 4 wks (18.8°). TUG performance improved (8.4 to 7.2 s, *P* < 0.05) and remained at end of program (7.6 s). Significant increase in concentric and eccentric torque of knee flexors and extensors.	The knee flexor stretching program was effective in increasing the flexibility of this muscle group, increasing knee-flexor and extensor torque and improving functional mobility in older adults. Most improvements lasted at least 4 wks after the stretching ceased. The increase in knee ROM is probably a result of adaptation in the connective tissue caused after the knee-flexor program.	13/18

Johnson et al. 2007 [25]. USA. Experimental (Pre-post within subjects). **Focus** Calves.	To investigate the effects of a static calf MTU stretching program on ankle dorsiflexion ROM in healthy adult women 65 years and older.	*n* = 13. Age: 84 ± 4.7 yr. Female. **Inclusion criteria** >65 yr, healthy.** ** No evidence of lower extremity dysfunction (assessed by visual observation of gait). Less than 10° of passive ankle dorsiflexion ROM. Functionally independent.	Pre-post, 6 wks. No control group. **Intervention** Supervised stretching of left and right calf muscle tendon unit (MTU). Standing with shoes on, placed one foot in front of other in comfortable stepping stance with hands on chair. Leaned forward on front leg until stretch felt in rear leg. Held for 60 s and repeated 4x/leg, once daily, 5 days wk^−1^ for 6 wks. **Assessment** Passive ankle dorsiflexion ROM by goniometry. Measured prior to stretching protocol and 3 days after-protocol. Posttesting researcher blind to pretesting data. **Analysis** Paired *t*-test.	Dorsiflexion ROM increased significantly from −11.1° to 1.2° (*P* < 0.001).	A 6-wk stretching protocol significantly improved ankle dorsiflexion ROM in elderly females. The ROM improvements were maintained 3 days after the last day. Were able to demonstrate a lasting, or plastic change in calf MTU passive ROM.	10/18

Gajdosik et al. 2005 [26]. USA. RCT. **Focus** Calves.	(1) To examine the effects of an eight-week stretching exercise program on calf muscle length, and on their length extensibility and passive resistive force properties for older women with limited dorsiflexion ROM. (2) To examine the influence of the stretching program on three functional tests.	*n* = 19. Age: 65–89 yr. Female. Stretching group *n* = 10. Age: 73 ± 6.8 yr. Control Group. *n* = 9.75 ± 8.3 yr. Community-dwelling. **Inclusion criteria** Active dorsiflexion ≤10°. Had ability to relax calf muscles and tibialis anterior during passive movements of ankle. No history of orthopaedic or neurological disorders. Minimally to moderately active.	Pre-post 8 wks. **Stretching group** Held static stretch for 15 s, 10 repetitions (total 150 s) 3x/wk for 8 wks. **Control group** Did not exercise. **Assessments** (Measured barefoot). **Functional** Timed agility course (modification of timed up-and-go). Timed 10 m walk. Standing forward functional reach. **Passive** Kin-Com ankle-foot apparatus (dorsiflexion) Dorsiflexion range of motion. Passive-electric energy (EMG). Passive resistive forces. **Analysis** Univariate ANOVA, two-way MANOVA, Pillai's Trace, two-way ANOVA for repeated measures, and one-way ANOVAs.	Stretching group significantly increased maximal passive dorsiflexion angle (11.1 to 16.2°, *P* < 0.001) and full stretch ROM (37.1 to 50.0°, *P* = 0.005). No significant changes in control group. Stretching group showed significant improvement in timed agility course (18.26 to 16.88 s, *P* = 0.008) and 10 m walk (6.44 to 5.99 s, *P* = 0.030). No change in control group speed. No change in functional reach test for either group. Stretching group increased both absorbed passive-elastic energy and retained passive-electric energy. MVC: Stretching group increases 14%, while control group increased 3.5% (both nonsignificant).	An 8-week stretching program for short calf muscles of older women increased the maximal DF ROM which indicated increased length of the calf muscles. The stretching program also increased the length extensibility, passive resistive forces and stored and retained passive elastic energy of the calf muscles. These adaptations correspond with decreased agility course and 10m walk times.	19/24

Petty et al. 1999 [27]. USA. Time-series, quasi-experimental. **Focus** Calves.	(1) To examine the relationship between maximum ankle dorsiflexion ROM and an individual's ability to move the trunk posteriorly with fixed BOS. (2) To examine intervention designed to increase gastrocnemius length on maximal ankle DF ROM and the ability to move posteriorly with fixed BOS. (3) To examine the contributions of maximum ankle DF ROM, age, and height to performance of volitional posterior trunk movement.	*n* = 7. 82 ± 4.5 yr. 4 male, 3 female. From audience at retirement community lecture. **Exclusion criteria** Any current neurological symptoms, taking any medications that might affect balance, any visual problems interfering with daily function, significant pain during backward lean test. **Inclusion criteria** >65 yr, no greater than 0° ankle DF ROM, at least 90° of shoulder flexion and 0° of elbow extension, ability to stand without external support for 2 min and perform Backward Lean Test properly, and not currently receiving physical therapy.	**Intervention** 4 wk stretching program: gastrocs stretching in “step-standing” position; point of stretch held for 30 s followed by 15 s rest; 4 repetitions per body part, repeated twice per day. **Assessments** Maximal ankle dorsiflexion (knee extended). Backward Lean Test (barefoot). **Analysis** Pearson product moment. correlation coefficient. Paired *t* test. Multiple regression analysis.	Mean pretest DF ROM: −5.9°± 2.5° increased to 0.5°± 2.9° (significant mean change of 6.4°± 2.2°) Backward Lean pre-test: 5.2 ± 3.5 cm increased to 9.3 ± 3.6 cm (significant mean change of 4.1 ± 2.2 cm).	The Backward Lean test proved useful for assessing changes in dynamic postural control in a posterior direction. The positive correlation between maximal ankle DF ROM and distance of posterior horizontal trunk excursion during Backward Lean may be related to the biomechanical requirements of the BL test. Subjects who performed an exercise program designed to lengthen the gastrocsoleus muscles demonstrated increased range of maximal ankle DF with knee extended and improved ability to lean backward while maintaining a stationary BOS. The significant increase in DF ROM from pretest to posttest supports the efficacy of the stretching protocol. A significant relationship exists between the degree of available maximal ankle DF ROM with the knees extended and the horizontal distance an individual is able to move the trunk posteriorly while maintaining a fixed BOS.	

Kerrigan et al. 2003 [28]. USA. Double-blind RCT. **Focus** Hip flexors.	To test the effect of a hip flexor stretching program on age-related gait changes about the ankle.	*n* = 96. Males = 30. Females = 66. Treatment. *n* = 47. 15 male, 32 women. Control. *n* = 49. 15 men, 34 women. **Inclusion criteria** ≥65 yrs old, healthy.	Pre-post 10 wks. **Protocol** Home exercises. Both groups performed warm-up and cool-down; 30 s stretches alternating limbs for 4 sets/8 stretches in total. 2x per day (approximately 5 min). **Treatment group** Hip stretching exercise. **Control group** Deltoid stretching exercise. **Assessment** Static hip extension range, goniometer. Pelvic and bilateral lower-extremity joint motion and joint kinematics. **Analysis** Unpaired and paired *t *tests, paired Student *t *tests, and Bonferroni adjustment.	Only peak ankle dorsiflexion and plantar flexion during swing increased significantly with treatment. Trend for increased in static peak hip extension (6.1 to 7.7°, *P* = 0.32). No major changes with control group. Both groups had similar minor increases in comfortable walking speed.	Tendency toward a reduction in anterior pelvic tilt implies that modest improvement in hip extension range allowed for a slight decrease in anterior pelvic tilt. These findings support the hypothesis that an increase in pelvic tilt in the elderly is a compensation for hip contracture rather than a compensation or a direct result of another impairment. Improvement in peak ankle plantar flexion with hip stretching and a trend toward improved ankle plantar flexor power at comfortable walking speed were found. These results suggest that these outcomes in the elderly may be secondary to proximal impairment rather than to impairment at the ankle or ankle musculature per se.	17/24

Rider & Daly 1991 [29]. USA. RCT. **Focus** Trunk.	To determine if a flexibility training program could positively influence the spinal mobility of older adults and be conducted in such a way as to facilitate the adoption of such a program by the participants as part of their weekly exercise routine.	*n* = 20. Females. Experimental group. *n* = 10. Control group. *n* = 10. Age: mean 72 yr. **Inclusion criteria** No orthopaedic conditions for example, moderate to severe back pain, no osteoporosis or any spinal deconditioning disorders.	Pre-post 10 wks. **Experimental group** Supervised flexibility exercises (sit and reach, knee tuck, pelvic lift, and back extension) 3 times each, held for 10 s each 3 days wk^−1^. **Control group** Continued current exercise program without flexibility training. **Assessments** Spinal flexion (sit and reach) Spinal extension. **Analysis** Repeated measures ANOVA.	Experimental group showed significant improvement (*P* < 0.05) pre to post for both spinal flexion (28.36 cm to 32.57 cm) and spinal extension (17.87 cm to 25.04 cm).	There is a meaningful positive association between regular flexibility training and spinal mobility in older population studies and may reduce the potential for age-related spinal deconditioning.	14/24

Weng et al. 2009 [30]. Taiwan. RCT (blind assignment). **Focus** Thighs.	To compare the effects of various stretching techniques on the outcomes of isokinetic exercise in patients with knee osteoarthritis (OA).	*n* = 132. *n* = 33 in each of 4 groups. Age: 64 ± 7.5 yr. Males = 26, Females = 106. **Inclusion criteria** Bilaterial moderate knee OA (Altman Grade II). No hip joint OA or any other hip problems with ROM limitations.	Pre-post 8 wks. **Intervention** **isokinetic exercise** 3x wk^−1^ for 8 wks at increasing doses. Active-assisted quadriceps and biceps femoris, holding end-point for 30 s, repeat 10x (10 min). *PNF*: HR(hold-relax), CR (contract-relax), CRAC (contract-relax agonist contract), HRAC (hold-relax agonist contract). Group 1: isokinetic muscular strengthening. Group 2: bilateral knee static stretching before isokinetic exercise. Group 3: PNF before isokinetic exercise. Group 4 (control): warm-up only. All groups received 10 min (15 min in control) hot packs and passive ROM on stationary bike. **Assessment** *ROM*: goniometer changes in knee active assisted ROM (flexion and extension). *Pain*: VAS scale. *Disability*: Lequesne's index (LI). *MPT*: flexion and extension with isokinetic dynamometer **Analysis** Weighted kappa statistics, Paired *t *tests, one-way ANOVA with Tukey's test, and Dunnett's test.	*n* = 124. Follow-up *n* = 111. *ROM*: significantly increased in groups II and III. *Pain: *decreased significantly in all groups, but increased in control. Greater decreases in II and greatest in III. *Disability: *average LI scores decreased significantly in all groups, greatest in III. *MPT:* average at 60°/second increased significantly in all measurements for groups II and III. All groups increased significantly more than control.	Stretching therapy is recommended as an adjuvant treatment to isokinetic exercise for patients with knee OA. PNF is more effective than static stretching exercise.	13/24

Yr: year; hr: hour; wk: week; wks: weeks; min: minutes; s: seconds; m: meters; ROM: range of motion; x: times; mo: months; pt: point; HRR: heart rate reserve; cm: centimeters.

**Table 2:** Sample electronic database search strategy.

	Sample search strategy-PubMed-January 2011	
(1)	Aged	331 611
(2)	Aging	236 732
(3)	Ageing	247 317
(4)	“Older age”	15 622
(5)	“Older adult*”	2 357
(6)	Elderly	3352095
(7)	Senior*	22 086
(8)	“Senior citizens”	734
(9)	(1 OR 2 OR 3 OR 4 OR 5 OR 6 OR 7 OR 8)	3505009
(10)	“Muscle stretching exercises” [Mesh]	420
(11)	“Pliability/physiology” [Mesh]	28
(12)	“Range of motion, Articular/physiology” [Mesh]	8 786
(13)	“Joint motion”	1 420
(14)	“Joint movement”	722
(15)	“Joint mobility”	888
(16)	“Joint range”	491
(17)	“Joint adhesion”	1 825
(18)	“Joint articulation”	42
(19)	“Muscle lengthening”	141
(20)	“Muscle elongation”	35
(21)	“Proprioceptive neuromuscular facilitation”	117
(22)	“Isometric contraction”	12 414
(23)	Yoga	1 483
(24)	“Tai chi”	455
(25)	Pilates	56
(26)	(10 OR 11 OR 12 OR 13 OR 14 OR 15 OR 16 OR 17 OR 18 OR 19 OR 20 OR 21 OR 22 OR 23 OR 24 OR 25)	28 177
(27)	Ability	455 298
(28)	Mobility	94 157
(29)	Frailty	2 112
(30)	Disability	80 338
(31)	Dependen*	1066172
(32)	Independen*	560 960
(33)	Reliance	8 424
(34)	Living	818 389
(35)	Institutionaliz*	12 329
(36)	“Nursing home”	13 615
(37)	“Activities of daily living”	45 277
(38)	“Independent activities of daily living”	3 979
(39)	ADL	4 683
(40)	IADL	1 147
(41)	“Assisted living”	1 197
(42)	“Long-term care”	25 605
(43)	“Long-term care”	25 605
(44)	(27 OR 28 OR 29 OR 30 OR 31 OR 32 OR 33 OR 34 OR 35 OR 36 OR 37 OR 38 OR 39 OR 40 OR 41 OR 42 OR 43)	2738402
(45)	Control	2295628
(46)	Treatment	6813000
(47)	Modality	165 777
(48)	Adjunct	25 544
(49)	Component	289 484
(50)	Group	1741947
(51)	Subset	76 495
(52)	Subset	446
(53)	Subgroup	55 099
(54)	Subgroup	2 358
(55)	“Exercise” [Mesh]	52 144
(56)	“Exercise prescription”	772
(57)	“Exercise program*”	3 376
(58)	“Exercise treatment”	240
(59)	“Exercise therapy”	20 527
(60)	Activity	1731305
(61)	“Physical activity”	37 516
(62)	“Physical therapy”	38 673
(63)	“Fitness program*”	277
(64)	Dance	2 274
(65)	“Rhythmic exercise”	86
(66)	(45 OR 46 OR 47 OR 48 OR 49 OR 50 OR 51 OR 52 OR 53 OR 54 OR 55 OR 56 OR 57 OR 58 OR 59 OR 60 OR 61 OR 62 OR 63 OR 64 OR 65)	9 702 261
(67)	(9 AND 26 AND 44 AND 66)	2 401
	(67 NOT child NOT children NOT cognit* NOT “alzheimer disease”	434
	NOT depression NOT anxiety NOT post-surg* NOT postsurg*	
	NOT surgery NOT surgical NOT cardiac NOT fracture* NOT arthroplasty	
(68)	NOT diabetes NOT diabetic NOT cancer NOT stroke NOT Parkinson*	
	NOT “multiple sclerosis” NOT sclerosis NOT stenosis NOT memory	
	NOT mental NOT coronary NOT cerebral NOT dystrophy NOT polio	
	NOT fibromyalgia)	

## Appendix

See Table 2.

## Abbreviations

ACSM: American College of Sports Medicine
RCT: Randomized control trial
TUG: Time-Up-and Go
ROM: Range of motion
PNF: Proprioceptive neuromuscular facilitation
CR-PNF: Contract-relax proprioceptive neuromuscular facilitation
EMG: Electromyography
ACR-PNF: Active assisted contract-relax proprioceptive neuromuscular facilitation.
